# Computational Experiments on the Step and Frequency Responses of a Three-Axis Thermal Accelerometer

**DOI:** 10.3390/s17112618

**Published:** 2017-11-14

**Authors:** Yoshifumi Ogami, Naoya Murakita, Koji Fukudome

**Affiliations:** 1 Department of Mechanical Engineering, College of Science and Engineering, Ritsumeikan University, 1-1-1 Noji-Higashi, Kusatsu, Shiga 525-8577, Japan; fukudome@cfd.ritsumei.ac.jp; 2 Murata Manufacturing Co., Ltd., Kyoto 617-8555, Japan; nao.32119@gmail.com

**Keywords:** thermal accelerometer, computational fluid dynamics, cross-axis sensitivity, step response, frequency response, nonlinearity

## Abstract

The sensor response has been reported to become highly nonlinear when the acceleration added to a thermal accelerator is very large, so the same response can be observed for two accelerations with different magnitudes and opposite signs. Some papers have reported the frequency response for the horizontal acceleration to be a first-order system, while others have reported it to be a second-order system. The response for the vertical acceleration has not been studied. In this study, computational experiments were performed to examine the step and frequency responses of a three-axis thermal accelerometer. The results showed that monitoring the temperatures at two positions and making use of cross-axis sensitivity allow a unique acceleration to be determined even when the range of the vertical acceleration is very large (e.g., −10,000–10,000 g). The frequency response was proven to be a second-order system for horizontal acceleration and a third-order system for vertical acceleration.

## 1. Introduction

Thermal accelerometers have recently attracted much attention and become the subject of theoretical, numerical, and experimental studies. Because thermal accelerometers do not have a proof mass, they can endure a higher shock than accelerometers that do have a proof mass. With a proof mass, a squeeze film effect is caused between it and the accelerometer structure, which requires a complex package to be removed.

**Figure 1:**
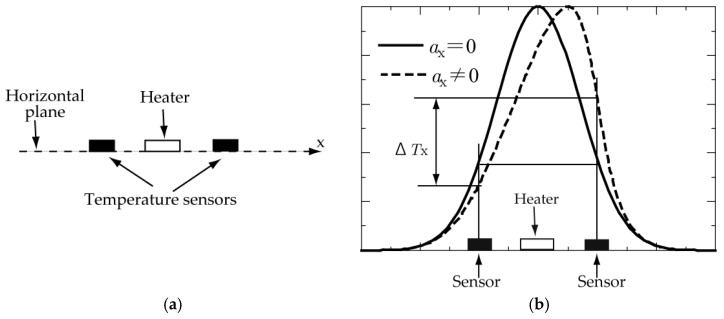
Principle to detect *x–y* (horizontal) accelerations: (**a**) heater and temperature sensors; (**b**) temperature profiles with and without acceleration.

A thermal accelerometer is based on the displacement of a hot air bubble generated by a heated wire in an enclosed chamber under acceleration. The principle of detecting *x–y* (i.e., horizontal) accelerations has been explained by others [1,2,3,4]. In short, two temperature sensors are positioned in parallel on a horizontal plane, and a heater is placed between them (Figure 1a). When no acceleration is applied to the accelerometer, the heater creates a symmetric heat bubble so that the same temperature is obtained by the two temperature sensors (Figure 1b). However, when a non-zero value of acceleration *a*_X_ in the *x*-direction is applied to the accelerometer, the shape of the heat bubble is distorted by the buoyancy effect. Consequently, the temperature difference *ΔT*_X_ is detected (Figure 1b). By knowing the relation between *a*_X_ and *ΔT*_X_ in advance, the arbitrary value of the acceleration added to the accelerometer can be calculated from the measured temperature difference *ΔT*_X_. The acceleration in the *y*-direction can be measured in a similar manner.

**Figure 2:**
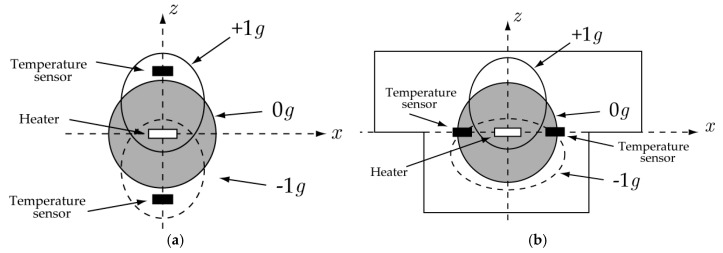
Principle to detect *z* (vertical) accelerations: (**a**) symmetric and (**b**) asymmetric structures.

To measure the acceleration in the *z*-direction (i.e., vertical direction), a few methods are available in the literature. A straightforward and simple method is to place the heater and temperature sensors on a vertical or inclined plane [5]. The second method is shown in Figure 2a; the heater and two temperature sensors are placed on three layers the same distance apart [6]. When neither acceleration nor gravity is applied to the accelerometer, a spherical heat bubble is created (i.e., the gray-filled circle), and the two sensors show the same temperature. As an example, however, if an acceleration of +1 g in the positive *z*-direction is applied, the heat bubble moves in the positive *z*-direction owing to the buoyancy effect (i.e., ellipse with solid line). Thus, the two sensors detect different temperature values, and the acceleration is determined by the temperature difference. The disadvantage of this method is that it takes up more space than the next method described below.

Figure 2b shows the third method. A cavity structure is created that is asymmetric in the up and down directions [7,8,9,10], and heat and temperature sensors are placed on the same plane. Because the shape of the deformed heat bubble varies according to the magnitude and sign of the acceleration in the *z*-direction, the measured temperature can determine the magnitude and sign of the acceleration. When the acceleration is very large, however, Nguyen et al. [10] pointed out that “the sensor response is highly non-linear,” such that “the same response can be observed for two accelerations with different magnitudes and opposite signs.” Therefore, it is impossible to determine which acceleration is correct when this response is observed with an accelerometer. The first purpose of this study was to address this difficulty by making use of cross-axis sensitivity, which we demonstrated in a computational experiment. 

Courteaud et al. [3] treated the frequency response of the accelerometer for the horizontal acceleration as a first-order system. On the other hand, Silva et al. fitted this frequency response to the curve of a second-order system (Figure 4 in [11]). Garraud et al. [4] and Nguyen et al. [10] studied the frequency response of the accelerometer but did not refer to the order of the system. Furthermore, none of the above studies considered the response for vertical acceleration. The second purpose of this study was to demonstrate, through a computational experiment, that the frequency response is a second-order system for horizontal acceleration and a third-order system for vertical acceleration. However, in this study, we did not try to improve the sensitivity of our thermal accelerometer, and we did not examine the effect of nonlinearity on the sensitivity.

## 2. Steady-State Step Response

### 2.1. Computational Model of the Accelerometer

**Figure 3:**
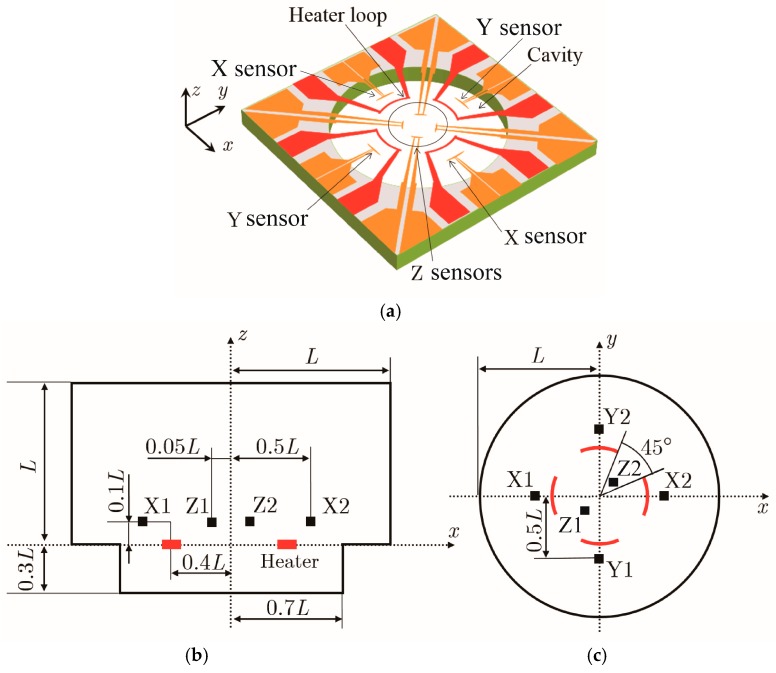

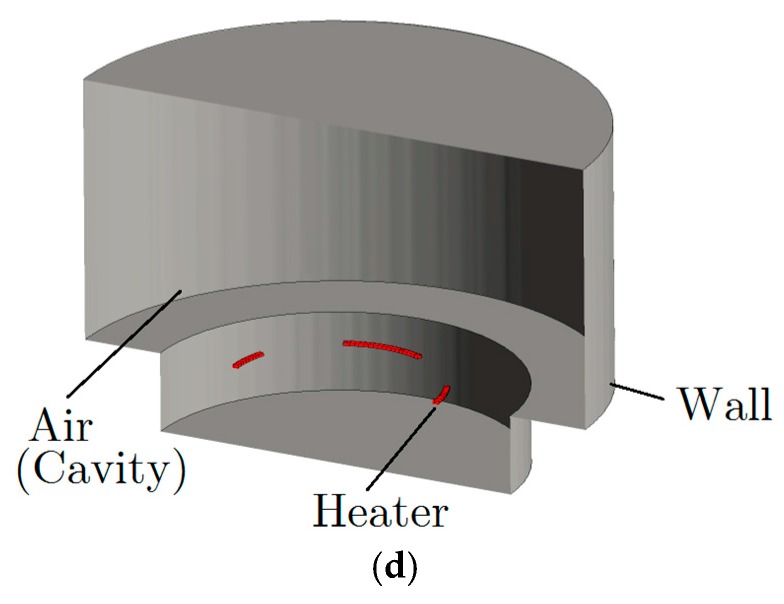
Computational model of the accelerometer: (**a**) 3D view; (**b**–**d**) side, top, and isometric views.

Figure 3a shows the 3D view of our model for the computational experiment, and Figure 3b–d respectively show the side, top, and isometric views of the simplified schematic. The cavity structure was asymmetric in the up and down directions as in [7,8,9,10]. The heater loop consisted of four circular arcs subtending 45° and placed on a circle with a radius of 0.4 *L*, where *L* is the reference length. The temperature sensors X1 and X2 for detecting acceleration in the *x*-direction and Y1 and Y2 for detecting acceleration in the *y*-direction were located outside the heater loop and, importantly, at a small elevation from the heater plane to obtain a better signal [7]. The sensors for the *z*-direction were located inside the heater loop. The positions of these sensors were found to be optimal based on a computational experiment that was performed in advance [7]. The device was packaged in a sealed chamber containing the working medium, in this case air (although helium can be used as well).

### 2.2. Equations and Computational Method

To capture the density–temperature dependence due to a large temperature change, the conservation laws of mass, momentum, and energy for a compressible fluid were employed to describe the thermofluidic phenomena in the accelerometer. These can be expressed as follows:

(1) $$\frac{\partial \rho }{\partial t}+\nabla \cdot (\rho u)=0,$$

(2) $$\frac{\partial (\rho u)}{\partial t}+u\cdot \nabla \cdot (\rho u)=-\nabla p+\nabla \cdot (\mu \nabla u),$$

and

(3) $$\frac{\partial (\rho c_{p}T)}{\partial t}+u\cdot \nabla \cdot (\rho c_{p}T)=\nabla \cdot (\lambda \nabla T),$$

where *u*, *p*, and *T* are the velocity vector, pressure, and temperature, respectively, of the fluid flow, and ρ, $c_{p},$ μ, and λ are the density, specific heat, viscosity, and thermal conductivity, respectively, of the fluid. To close the equations, we assumed that the working fluid obeys the ideal gas law for compressible flows:

(4) $$p=\frac{\rho RT}{M},$$

where *R* is the universal gas constant, and *M* is the molar mass of the gas.

**Figure 4:**
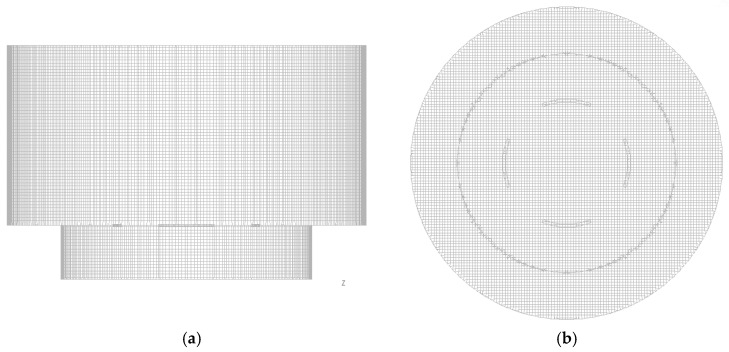
Computational mesh: (**a**) side and (**b**) top views.

The computation region of the accelerometer was decomposed into a hexagonal mesh, as shown in Figure 4, with the mesh generation software ANSYS ICEM CFD. The mesh number was 674,015, which is large enough to obtain accurate solutions [7]. Flow parameters such as *u*, *p*, and *T* for this mesh were then obtained with the computational fluid dynamics package ANSYS Fluent, and the finite volume method was used to discretize the governing Equations (1)–(4). The SIMPLEC method was adopted for pressure–velocity coupling, and all spatial discretizations were performed with the second-order center scheme. The heaters were simulated as a solid zone with a constant heat generation rate.

### 2.3. Conditions and Results

For the computational experiment, the length L (Figure 3) was set to 1 mm, and the temperatures on the wall and heater were respectively kept at 300 K and 500 K. The working medium was air. The accelerations in the x-direction $a_{X},$ and y-direction $a_{Y}$ that were added to the accelerometer were 0 g, +1 g, +2 g, +3 g, +4 g, +5 g, +10 g, +20 g, +50 g, +100 g, and +200 g. Only positive values were adopted because the temperature measured at temperature sensor X2 (Figure 3) for positive acceleration in the x-direction was the same as that measured at temperature sensor X1 for negative acceleration in the x-direction owing to the symmetric structure in both the x- and y-directions. Thus, the response to the negative accelerations could be obtained by the response to the positive accelerations. On the other hand, the accelerations in the z-direction $a_{Z}$ were set to both positive and negative values: 0 g, ±1 g, ±2 g, ±3 g, ±4 g, ±5 g, ±10 g, ±20 g, ±50 g, ±100 g, and ±200 g. This was because of the asymmetric structure in the z-direction. For all computational experiments, these constant accelerations were suddenly added at time 0 to the accelerometer under no acceleration (time <0). Only the steady state of the step response was considered.

**Figure 5:**
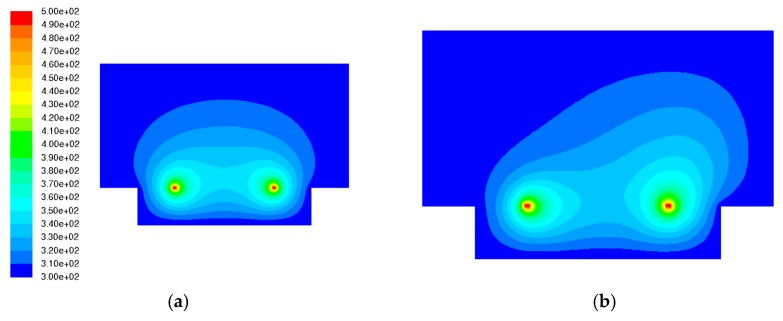
Temperature contours on the *x–z* plane: (**a**) $\left(a_{X},\;a_{Y},\;a_{Z})=(0g,\;0g,\;0g\right)$; (**b**) $\left(a_{X},\;a_{Y},\;a_{Z})=(200g,\;0g,\;0g\right)$.

Figure 5 shows two examples of temperature contours on the *x–z* plane when (a) no acceleration or gravity was added to the accelerometer and (b) an acceleration of 200 g was added only in the *x*-direction. The heat bubble that was symmetric under no acceleration showed remarkable deformation with a large acceleration due to the buoyancy effect. We defined $\Delta T_{X1}$ and $\Delta T_{Z1}$ as the differences between temperatures measured by temperature sensors X1 and Z1, respectively, with gravity only and with the added acceleration.

**Figure 6:**
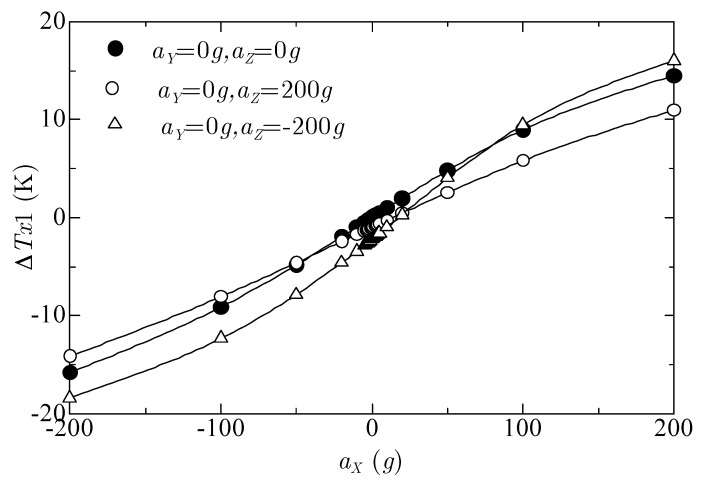
Relations between $\Delta T_{X1}$ and *x*-acceleration $a_{X}$, with *y*-acceleration $a_{Y}=0g,$ and with *z*-accelerations $a_{Z}=0g,\;200g$, and −200 g.

Figure 6 shows the relation between the temperature difference $\Delta T_{X1}$ and *x*-acceleration $a_{X},$ with the *y*-acceleration $a_{Y}=0g$, and with three *z*-accelerations $a_{Z}=0g,\;200g$, and −200 g. The absolute value of $\Delta T_{X1}$ increased but not linearly with the absolute value of the *x*-acceleration $a_{X}$. The three curves were not on one curve because of the cross-axis sensitivity of the *z*-acceleration affecting $\Delta T_{X1}$. As shown below, this sensitivity is not a problem when obtaining the acceleration from the temperature difference.

**Figure 7:**
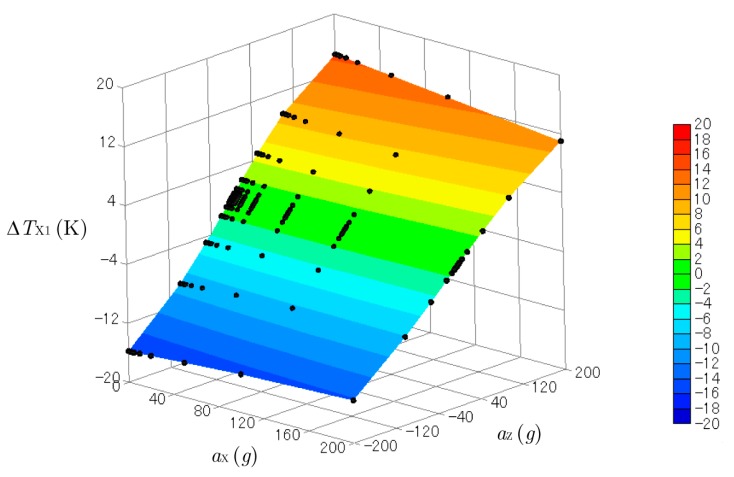
Three-dimensional plot of $\Delta T_{X1}$ created by the accelerations of $a_{X}$ (from 0 g to 200 g) and $a_{Z}$ (from −200 g to 200 g).

**Figure 8:**
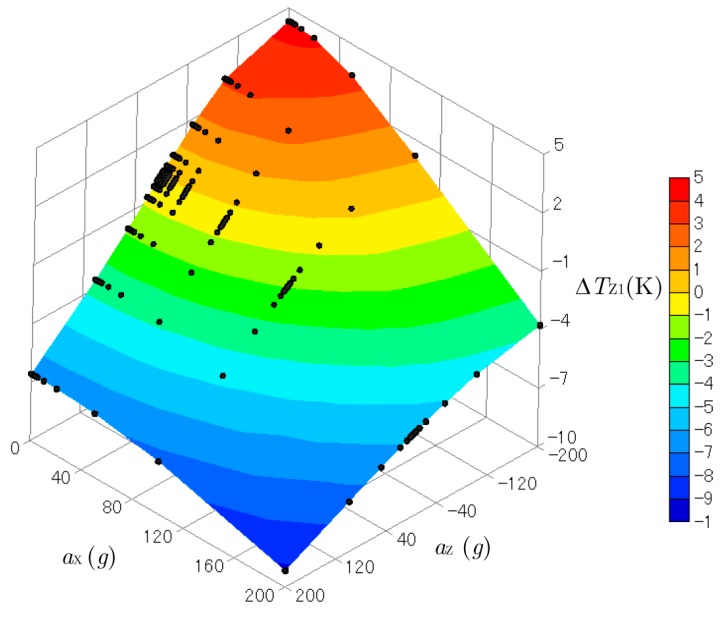
Three-dimensional plot of $\Delta T_{Z1}$ created by the accelerations of $a_{X}$ (from 0 g to 200 g) and $a_{Z}$ (from −200 g to 200 g).

Figure 7 and Figure 8 show three-dimensional plots of $\Delta T_{X1}$ and $\Delta T_{Z1}$ produced by the accelerations combined with $a_{X}$ (from 0 to 200 g) and $a_{Z}$ (from −200 to 200 g)*.* The *y*-acceleration was kept at $a_{Y}=$ 0 g. The black dots on the curved surfaces represent the data obtained by the computational experiments. These surfaces were not flat, so the relation between the outputs $\Delta T_{X1}$ and $\Delta T_{Z1}$ and the inputs $a_{X}$ and $a_{Z}$ were not linear. Cross-axis sensitivity was also observed. The temperature difference in the *x*-direction $\Delta T_{X1}$ obtained by the sensor for the *x*-acceleration was affected by the *z*-acceleration, and $\Delta T_{Z1}$ was affected by the *x*-acceleration. However, cross-axis sensitivity and nonlinearity are not a problem when calculating the acceleration from the measured temperature, as explained below.

**Figure 9:**
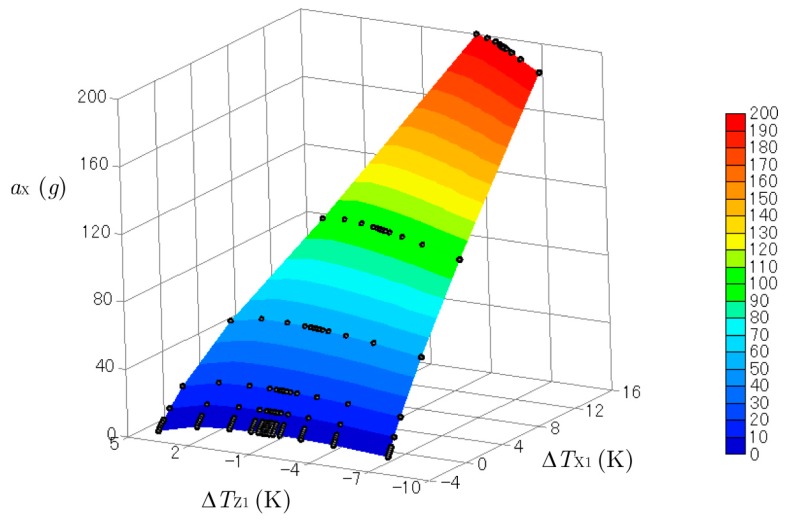
*x*-acceleration *a*_X_ calculated from $\Delta T_{X1}$ and $\Delta T_{Z1}.$

**Figure 10:**
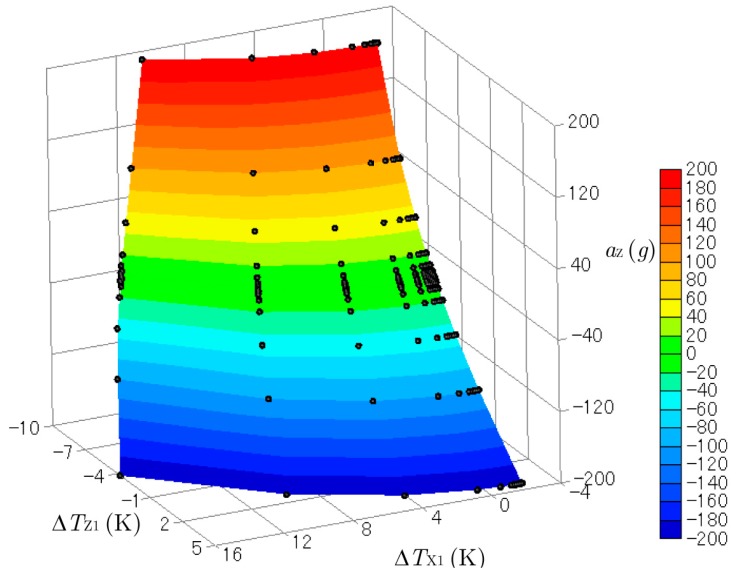
*z*-acceleration *a*_Z_ calculated from $\Delta T_{X1}$ and $\Delta T_{Z1}.$

Figure 9 and Figure 10 show the inverse of Figure 7 and Figure 8. When the accelerometer is accelerated in both the *x-* and *z*-directions by unknown values, the temperature differences $\Delta T_{X1}$ and $\Delta T_{Z1}$ are measured by the *x*- and *z*-sensors, respectively. Then, the *x*- and *z*-accelerations $a_{X}$ and $a_{Z}$ can be calculated by interpolation using the data shown in Figure 9 and Figure 10. Thus, by using the two output values of $\Delta T_{X1}$ and $\Delta T_{Z1}$, the input (unknown) accelerations $a_{X}$ and $a_{Z}$ can be obtained even with cross-axis sensitivity and nonlinearity.

Next, we focus on the problem discussed in Section 1 and by Nguyen et al. [10]. The acceleration considered exclusively for this problem is *a*_Z_. However, the range is very large, insofar as *a*_Z_ = −10,000 g to 10,000 g, as treated by Nguyen et al. [10]. The combined accelerations of the three components—*a*_X_ and *a*_Z_, or *a*_X_, *a*_Y_, and *a*_Z_—are not considered, because doing so requires a considerable number of computer simulations. Moreover, it suffices only to change the *z*-acceleration for the purpose of studying this problem.

**Figure 11:**
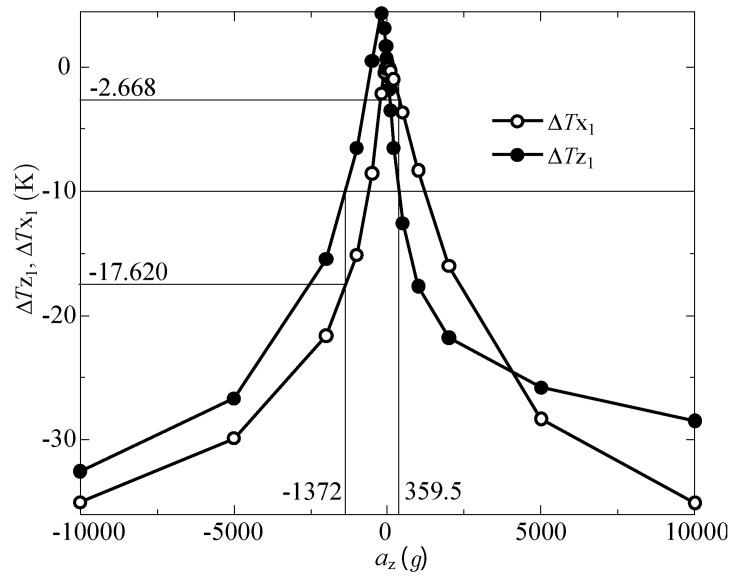
Temperature differences measured by the *x*-sensor $\Delta T_{X1}$ and *z*-sensor $\Delta T_{Z1}$ for a large *z*-acceleration range (*a*_Z_ = −10,000 g to 10,000 g) with *a*_X_ = *a*_Y_ = 0 g.

Figure 11 shows the computational result for the temperature differences measured by the *z*-sensor, $\Delta T_{Z1}$ (filled black circles), and the x-sensor, $\Delta T_{X1}$ (unfilled black circles), for a large *z*-acceleration range of *a*_Z_ = −10,000 g to 10,000 g, with *a*_X_ = *a*_Y_ = 0 g. The measured temperature difference, which is given by $\Delta T_{Z1}=-10$ K as an example, can be produced by two accelerations: −1372 g and 359.5 g. Therefore, it is impossible to determine which acceleration is correct when $\Delta T_{Z1}=-10$ K is measured with this accelerometer. As pointed out by Nguyen et al. [10], when the acceleration is very large, “the sensor response is highly non-linear” so “the same response can be observed for two accelerations with different magnitudes and opposite signs.” Consequently, they considered only a smaller measurement range up to ±5g, for which good linearity was observed. However, this problem can be solved as explained below.

In Figure 11, the other temperature difference $\Delta T_{X1}$ produced by cross-axis sensitivity takes two different values of −17.620 K and −2.668 K for each *a*_Z_. If there were no cross-axis sensitivity, $\Delta T_{X1}$ would take a zero value when only the *z*-acceleration was added to the accelerometer. Therefore, by monitoring both $\Delta T_{X1}$ and $\Delta T_{Z1}$—in other words, by making use of cross-axis sensitivity—we can determine which acceleration value is correct even when the *z*-acceleration range is very large and the sensor response is highly nonlinear. 

**Figure 12:**
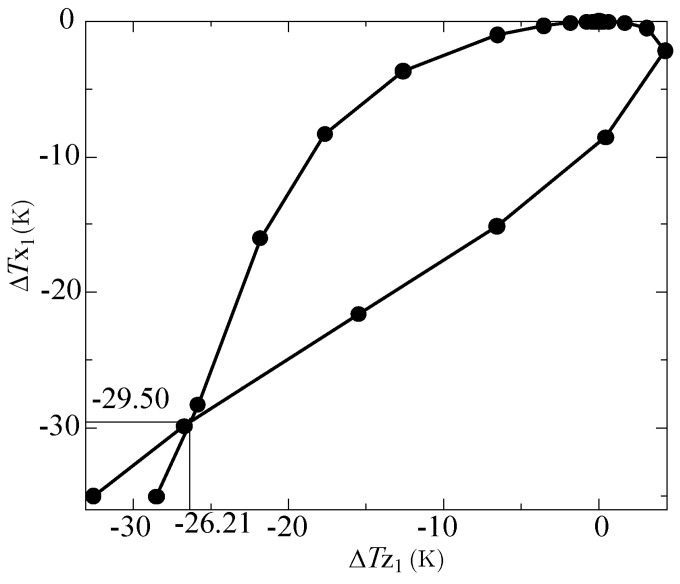
Relation between $\Delta T_{X1}$ and $\Delta T_{Z1}$.

Note that another problem arises when graphing the relation between $\Delta T_{X1}$ and $\Delta T_{Z1}$, as shown in Figure 12. The curve intersects at ($\Delta T_{Z1}$, $\Delta T_{X1}$) = (−26.21 K, −29.50 K), where the acceleration values are *a*_Z_ = −4864 g and 5855 g. This makes it impossible to determine what acceleration produced these measured temperature differences. Fortunately, this problem can easily be solved by changing the position of the *z*-sensor, as explained below.

**Figure 13:**
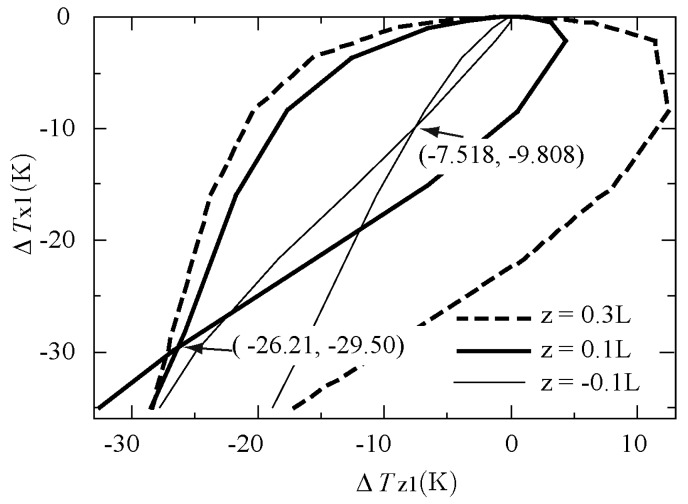
Relation between $\Delta T_{X1}$ and $\Delta T_{Z1}$ for three *z*-sensor positions.

For the above computational experiments, the height of the *z*-sensor was 0.1 *L*, where *L* = 1 mm, as explained above. Figure 13 compares the results with three values for the height of the *z*-sensor: −0.1 *L*, 0.1 *L*, and 0.3 *L*. When the position of the *z*-sensor was lowered from 0.1 *L* to −0.1 *L*, the intersection point still existed and only shifted to the position ($\Delta T_{Z1}$, $\Delta T_{X1}$) = (−7.518 K, −9.808 K) (thin line). On the other hand, by elevating the position of the *z*-sensor from 0.1 *L* to 0.3 *L*, the intersection of the curve disappeared (dashed line). Thus, one combination of outputs $\Delta T_{Z1}$ and $\Delta T_{X1}$ can determine a single acceleration value of *a*_Z_, even when the *z*-acceleration range is very large (e.g., −10,000 g to 10,000 g) and the sensor response is highly nonlinear.

## 3. Frequency Response

Courteaud et al. [3] stated that “the frequency response of the accelerometer is function of two phenomena. The first one corresponds to the frequency response of the fluid to the acceleration induced by the difference of temperature gradient on the sensitive axis. The second one corresponds to the frequency response of the detector to the temperature variation in the fluid.” In this section, we present a computational experiment performed to examine the frequency response of the fluid to the acceleration and show that the frequency response is a second-order system for horizontal acceleration and a third-order system for vertical acceleration.

### 3.1. Frequency Response for Horizontal Acceleration

We performed a computational experiment to study the frequency response of our accelerometer for the model size *L* = 1 mm. The acceleration with the frequency *f* in the horizontal direction (*x*-direction) added to the accelerometer is given by

(5) $$a_{X}(t)=10g\;\sin(2\pi ft).$$

The amplitude was kept at 10 g (=98.1 m/s^2^), and the frequency *f* was varied from 1 Hz to 5000 Hz. The power of the heater was 15 mW, the wall temperature was 300 K, and *z*-acceleration was the same as gravitational acceleration −1 g.

The magnitude $M_{X}$ is defined by

(6) $$M_{X}=20\;\log_{10}\left(\frac{\Delta T_{\omega }}{\Delta T_{0}}\right),$$

where $\Delta T_{\omega }$ is the difference between the maximum and minimum temperatures produced at the temperature sensors by the sinusoidal acceleration given in (5), and $\Delta T_{0}$ is the temperature difference measured at sensors *T*_X1_ and *T*_X2_ for a step response with the *x*-acceleration *a*_X_ = 10 g. As the frequency decreased, $\Delta T_{\omega }$ became closer to $\Delta T_{0}$ until finally *M_X_*= 0.

**Figure 14:**
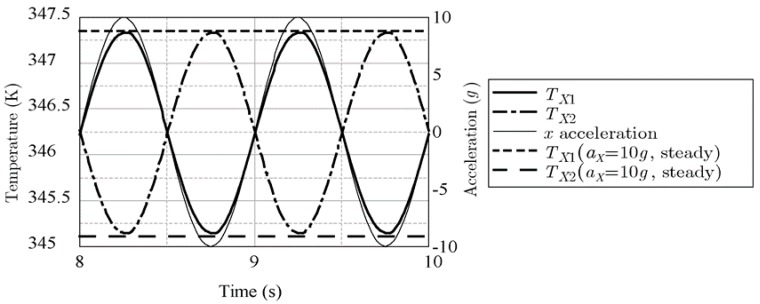
Frequency response by *x*-acceleration with ω = 1 Hz.

**Figure 15:**
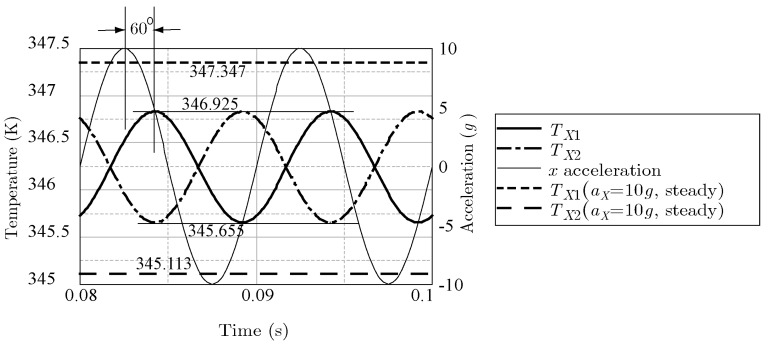
Frequency response by *x*-acceleration with ω **=** 100 Hz.

Figure 14 shows an example frequency response for *x*-acceleration with *f* = 1 Hz (thin line) measured by the two *X*-sensors *T*_X1_ and *T*_X2_ (the thick line and dot-and-dash line, respectively). The steady-state response with constant *x*-acceleration *a*_X_ = 10 g is also shown with a dashed line (*T*_X1_ = 347.347 K) and long dashed line (*T*_X2_ = 345.113 K). The maximum and minimum values of the frequency response with *f* = 1 Hz were almost the same as the steady-state values, which implies that *M_X_*= 0. The delay of the phase of the frequency response was small. On the other hand, Figure 15 shows that, when the frequency was increased to 100 Hz, the maximum and minimum values of the frequency response became 346.925 K and 345.655 K, respectively, and the delay of the phase shift was measured to be almost 60°.

**Figure 16:**
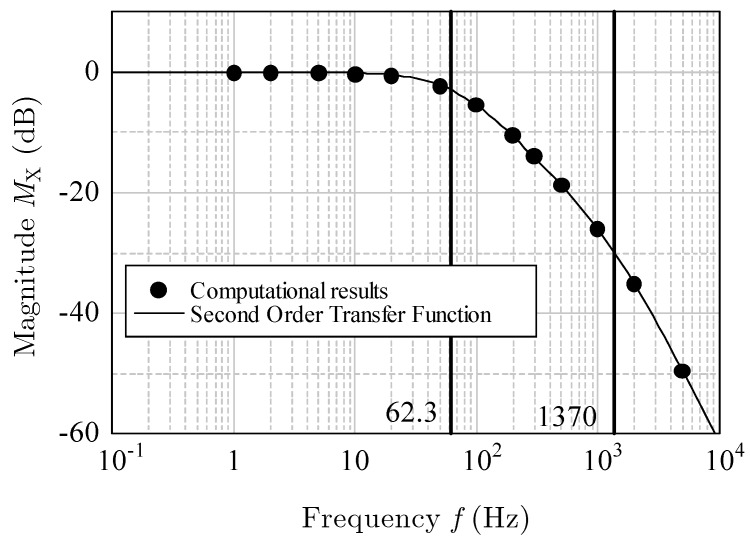
Magnitude *M*_X_ (dB) for *x*-acceleration.

**Figure 17:**
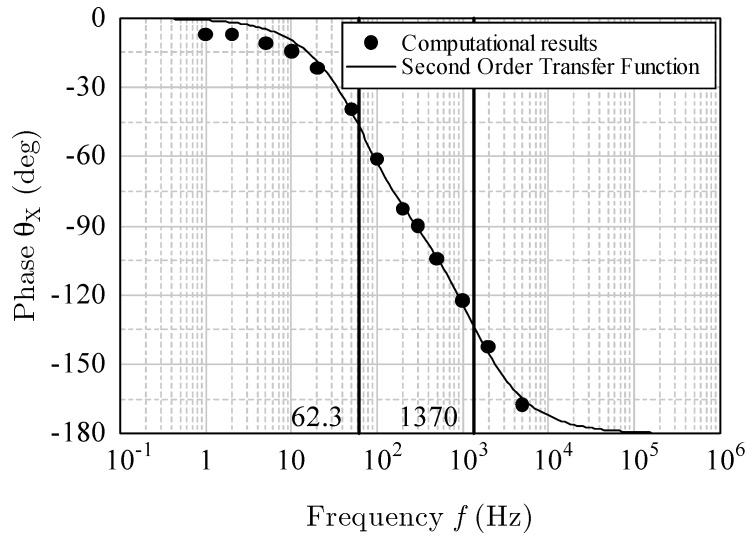
Phase shift for *x*-acceleration.

In Figure 16 and Figure 17, the dots indicate the computational results of the magnitude in decibels and the phase shift for the *x*-acceleration at various frequencies. Because the phase shift was from 0° to −180°, the *x*-acceleration was considered to have a second-order response ([12], pp. 10–34). Therefore, the transfer function can be written as

(7) $$G(s)=\frac{1}{\left(\frac{s}{\omega_{n1}}+1\right)\left(\frac{s}{\omega_{n2}}+1\right)},$$

where $\omega_{n1}$ and $\omega_{n2}$ are the corner frequencies (approximately 62.3 and 1370 Hz). The damper factor is 2.45. The solid lines in Figure 16 and Figure 17 indicate the magnitude $M_{X}$ in decibels, and the phase $\theta_{X}$ is given by

(8) $$M_{X}=20\;\log_{10}\left|G(j\omega )\right|,$$

(9) $$\theta_{X}=∠G(j\omega ).$$

The curves of the theoretical Equations (7)–(9) agreed well with the computational results. Thus, the frequency response for the horizontal acceleration was proved to be a second-order system.

### 3.2. Frequency Response for Vertical Acceleration

The acceleration with the frequency ω in the vertical direction (*z*-direction) added to the accelerometer is given by

(10) $$a_{Z}(t)=-g+10g\;\sin(2\pi ft)$$

**Figure 18:**
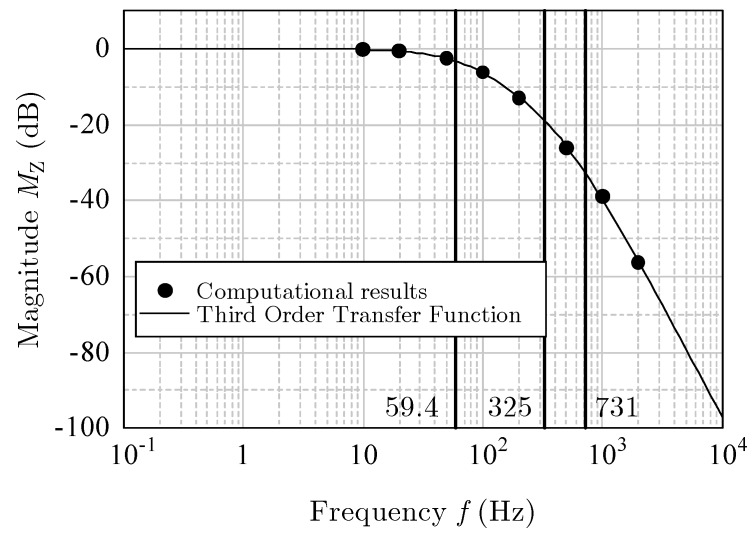
Magnitude *M*_X_ (dB) for *z*-acceleration.

**Figure 19:**
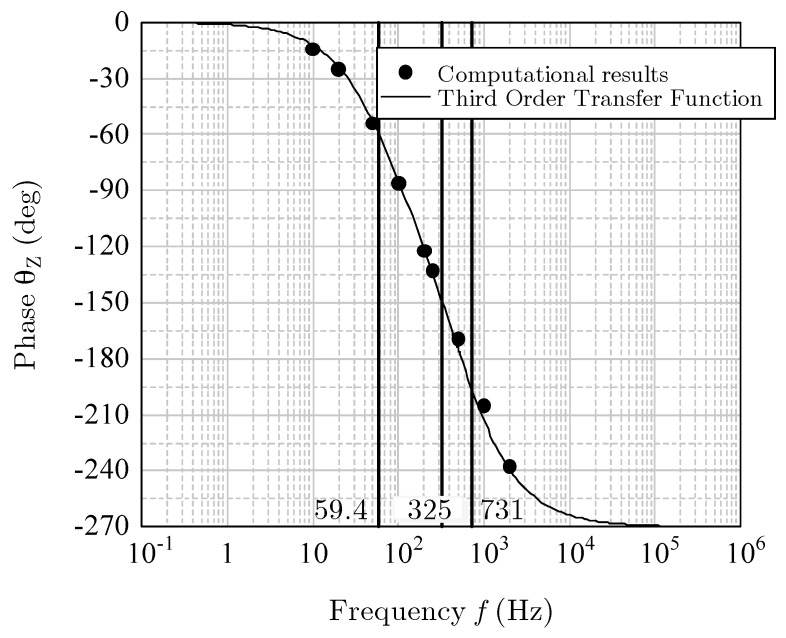
Phase shift for the *z*-acceleration.

As before, the amplitude was kept at 10 g (=98.1 m/s^2^), and the frequency *f* was changed from 10 Hz to 2000 Hz. Because the computational results of the phase shift were from 0° to −270°, the response was considered to be a third-order ([12], pp. 10–34). Therefore, the transfer function can be written as

(11) $$\begin{array}{l} G(s)=G_{1}(s)G_{2}(s)\\ \left\{ \begin{array}{l} G_{1}(s)=\frac{1}{\frac{s}{\omega_{n1}}+1}\\ G_{2}(s)=\frac{1}{\left(\frac{s}{\omega_{n2}}+1\right)\left(\frac{s}{\omega_{n3}}+1\right)} \end{array}\right. \end{array}$$

where $\omega_{n1}$, $\omega_{n2}$, and $\omega_{n3}$ are the corner frequencies of approximately 59.4, 325, and 731 Hz, respectively. The damper factor is 1.09. The solid lines in Figure 18 and Figure 19 indicate the magnitude $M_{Z}$ in decibels, and the phase $\theta_{Z}$ is given as follows:

(12) $$M_{Z}=20\;\log_{10}\left|G_{1}(j\omega )\right|+20\;\log_{10}\left|G_{2}(j\omega )\right|,$$

(13) $$\theta_{Z}=∠G_{1}(j\omega )+∠G_{2}(j\omega ).$$

The curves of the theoretical Equations (11)–(13) agreed well with the computational results. Thus, the frequency response for vertical acceleration was proved to be a third-order system.

## 4. Discussion and Conclusions

Section 2 introduced the principle to obtain a unique solution under the condition of a highly nonlinear sensor response with cross-axis sensitivity. Cross-axis sensitivity is not to be removed but rather exploited. For a thermal accelerometer, considering the temperature sensors for acceleration in only designated directions may be ineffective. By making data maps as shown in Figure 9 and Figure 10, two (or three) responses from two (or three) temperature sensors can determine a unique set of two (or three) components of acceleration. Indeed, this can be done even when the acceleration range is very large, when the sensor response is highly nonlinear, and when cross-axis sensitivity is observed. As such, thermal accelerometers can be used under more severe conditions.

In this study, we did not try to improve the sensitivity of our thermal accelerometer or examine the effect of nonlinearity on sensitivity. In addition, we did not study the step responses due to combined accelerations of the three components (viz., *a*_X_, *a*_Y_, and *a*_Z_ for short ranges; and *a*_X_ and *a*_Z_, or *a*_X_, *a*_Y_, and *a*_Z_ for very large ranges). As shown in Figure 12 and Figure 13, the ambiguity (intersection of the curve) was easily removed by changing the height of the *z*-sensor when only *z*-acceleration was added to the accelerometer. However, when adding both *x*- and *z*-acceleration to the accelerometer, it was found that the points of intersection increased. This can be overcome by changing the positions of the temperature sensors and/or increasing the number of temperature sensors. Because such research requires many computer simulations and analyses, however, this will be discussed in future research.

As noted at the beginning of Section 3, the frequency response of the accelerometer is a function of two phenomena [3]. However, only the first phenomenon was treated here: namely, the frequency response of the fluid to acceleration induced by a difference in the temperature gradient. Future work will involve studying the second phenomenon: namely, the frequency response of the detector to temperature variation in the fluid.

The main conclusions are summarized as follows:

1. By monitoring the temperatures at two positions and making use of cross-axis sensitivity, a unique acceleration can be determined even when the range of vertical acceleration is very large, such as −10,000–10,000 g.
2. Two (or three) responses from two (or three) temperature sensors can determine a unique set of two (or three) components of acceleration even when the acceleration range is very large, the sensor response is highly nonlinear, and cross-axis sensitivity is observed.
3. The frequency response for horizontal acceleration is a second-order system.
4. The frequency response for vertical acceleration is a third-order system.
